# Associations Between Genetic Polymorphisms and Psychological Variables in Women With Fibromyalgia: A Cross‐Sectional Study

**DOI:** 10.1002/ejp.70201

**Published:** 2025-12-29

**Authors:** Víctor Riquelme‐Aguado, María Elena González‐Álvarez, Silvia Di‐Bonaventura, Leonardo Rodríguez‐Lagos, Alazne Zabarte del Campo, Francisco Gómez‐Esquer, Gema Díaz‐Gil, Antonio Gil‐Crujera

**Affiliations:** ^1^ Department of Basic Health Sciences Rey Juan Carlos University Madrid Spain; ^2^ Research Group on Bases Anatómicas, Moleculares y del Desarrollo Humano (GAMDES) Faculty of Health Sciences Rey Juan Carlos University Madrid Spain; ^3^ Research Group on Cognitive Neuroscience, Pain, and Rehabilitation (NECODOR), Faculty of Health Sciences Rey Juan Carlos University Madrid Spain; ^4^ Health Science UNIE University Madrid Spain; ^5^ Department of Physical Therapy, Occupational Therapy, Rehabilitation and Physical Medicine Rey Juan Carlos University Alcorcón Spain; ^6^ Fundación Internacional para el Estudio y Tratamiento del Dolor PainCorp Foundation Madrid Spain

**Keywords:** anxiety, depression, fibromyalgia, genetic polymorphisms, kinesiophobia, pain catastrophising

## Abstract

**Introduction:**

Fibromyalgia (FM) is a multifactorial syndrome involving chronic pain and psychological distress. Psychological traits such as anxiety, depression, and catastrophising are linked to symptom severity. Genetic variability may contribute to these dimensions through mechanisms related to pain modulation and stress response.

**Objectives:**

To examine associations between selected genetic polymorphisms and psychological variables in women with FM.

**Methods:**

A cross‐sectional study was conducted in 67 women diagnosed with FM. Pain intensity, FM impact and psychological variables—anxiety, depression and catastrophising—were assessed using validated questionnaires. Saliva samples were collected and 10 SNPs were genotyped (*COMT* rs4680, *DRD3* rs6280, *OPRM1* rs1799971, *BDNF* rs6265, *MAOA* rs1137070, *FKBP5* rs1360780, *IL6* rs1800796, *TNF* rs1800629, *IL10* rs1800896, *IFITM3* rs12252). Correlations were assessed using Pearson or Spearman coefficients, and associations were examined using ANOVA or Kruskal–Wallis with Tukey or Mann–Whitney post hoc tests.

**Results:**

Pain intensity correlated with depression (*r* = 0.476, *p* < 0.001), catastrophising (*r* = 0.414, *p* < 0.001), and anxiety (*r* = 0.314, *p* = 0.009). Catastrophising was related to depression (*r* = 0.615, *p* < 0.001), anxiety (*r* = 0.453, *p* < 0.001), and kinesiophobia (*r* = 0.445, *p* < 0.001). *BDNF* rs6265 was associated with catastrophising (*p* = 0.044), *OPRM1* rs1799971 with anxiety (*p* = 0.030), and *MAOA* rs1137070 with depression (*p* = 0.020).

**Conclusions:**

Psychological variables in FM are interrelated and linked to pain perception. *BDNF*, *OPRM1* and *MAOA* polymorphisms are associated with indices of psychological vulnerability, underscoring the importance of integrating genetic and psychological perspectives to understand variability in FM.

**Significance Statement:**

Genetic variability influences psychological vulnerability in fibromyalgia. Specific variants were associated with key psychological traits: BDNF rs6265 with pain catastrophising, OPRM1 rs1799971 with anxiety, and MAOA rs1137070 with depressive symptoms. These findings reveal an interplay between genetic and psychological factors that may guide more personalised strategies for managing fibromyalgia.

## 1 Introduction

Fibromyalgia (FM) is a chronic and disabling syndrome, predominantly affecting women, and characterised by widespread pain, fatigue, cognitive dysfunction, and sleep disturbances (Wolfe et al. 2013). Although its aetiology remains unclear, evidence supports a multifactorial origin involving central sensitisation (Or‐Borichev et al. 2025), neuroendocrine dysfunction (Demori et al. 2024), and immune alterations (Nizzero et al. 2025). In this framework, psychological factors such as anxiety, depression, and pain catastrophising are increasingly recognised as integral to FM pathophysiology, shaping symptom expression and trajectory (Bushnell et al. 2013; Ellingson et al. 2018; González‐Álvarez et al. 2024; Ji et al. 2018; Karakuş et al. 2025; Malfliet et al. 2017).

Genetic polymorphisms have been investigated in FM for over a decade and continue to attract research interest (Janssen et al. 2021), including variants related to physiological mechanisms, such as increased central sensitisation, elevated neuroinflammatory activity, and altered stress response (D'Agnelli et al. 2019). However, the role of these SNP polymorphims in psychological traits—including stress reactivity, anxiety, or depression—remains underexplored despite their impact on quality of life (Janssen et al. 2021).

In this context, increasing interest in the genetic basis of FM and related conditions has led to the identification of several genes involved in key physiological mechanisms (Bernik et al. 2013). These variants—summarised in Table 1—include genes implicated in central pain perception, emotional regulation, and immune response, which are functional domains frequently altered in FM.

**TABLE 1:** Summary of selected single nucleotide polymorphisms (SNPs) analysed in this study, grouped by biological pathway. The table includes gene name, variant, biological function role, associated disorders and relevance to pain‐related conditions. References supporting SNP selection, based on their role in mechanisms relevant to fibromyalgia pathophysiology, were included.

Biological pathway	Gene	SNP	Mechanism	Main associated disorders	Role in pain‐related disorders	References
Central pain modulation	*COMT*	rs4680	Catecholamine metabolism	FM, chronic widespread pain, migraine and temporomandibular disorders	Associated with pain sensitivity and stress vulnerability	Fernández‐de‐las‐Peñas et al. 2012; Lami et al. 2018; Vetterlein et al. 2023
	*BDNF*	rs6265	Activity‐dependent BDNF secretion	FM, mood disorders and neuropathic pain	Influences emotional regulation and pain processing	Guillot et al. 2023; Zinchuk et al. 2024
	*OPRM1*	rs1799971	μ‐opioid receptor function	FM, chronic pain conditions	Modulates pain perception and anxiety	Estévez‐López et al. 2018
	*DRD3*	rs6280	Dopaminergic signalling	Chronic pain conditions, mood disorders	Linked to depression and pain modulation	Potvin et al. 2009
HPA axis regulation	*MAOA*	rs1137070	Monoamine degradation	FM, mood disorders and stress‐related pain	Linked to stress response	Gürsoy et al. 2008
	*FKBP5*	rs1360780	Glucocorticoid receptor sensitivity	Stress‐related pain, neuropathic pain	Linked to stress response	Hohne et al. 2015
Immune response	*IL6*	rs1800796	Pro‐inflammatory cytokine signalling	Rheumatoid arthritis, chronic inflammatory pain	Associated with inflammation enhancing nociceptive sensitisation	Zhou et al. 2016; Fernández‐de‐las‐Peñas et al. 2022; Zlendić et al. 2024
	*TNF*	rs1800629	Pro‐inflammatory cytokine; TNF‐α signalling	Rheumatoid arthritis, autoimmune pain disorders	Dysregulation may contribute to persistent pain	Braga et al. 2021; Fernández‐de‐las‐Peñas et al. 2022
	*IL10*	rs1800896	Anti‐inflammatory cytokine signalling	Rheumatoid arthritis, musculoskeletal pain	Linked to chronic inflammation and pain	Braga et al. 2021; Fernández‐de‐las‐Peñas et al. 2022
	*IFITM3*	rs12252	Neuroimmune signalling; inflammation; viral susceptibility	Virus‐induced inflammatory disorders	Role in neuroimmune interactions potentially amplifying pain	Gómez et al. 2021

Genes involved in nociception have been studied in several chronic pain conditions and emerging evidence suggests their importance in FM. The Catechol‐O‐Methyltransfere (*COMT*) rs4680 variant has been associated with increased pain sensitivity and stress vulnerability in FM populations (Fernández‐de‐las‐Peñas et al. 2012; Lami et al. 2018; Vargas‐Alarcón et al. 2007; Vetterlein et al. 2023). Brain‐Derived Neurotrophic Factor (*BDNF*) rs6265 variant has been linked to emotional regulation and vulnerability to mood disorders and FM (Guillot et al. 2023; Zinchuk et al. 2024). *OPRM1* rs1799971 polymorphism has been suggested to play a role in FM‐related analgesic response (Estévez‐López et al. 2018). Dopamine Receptor D3 (*DRD3*) rs6280 polymorphism has been associated with depressive symptoms (Potvin et al. 2009), although not specifically in FM.

Alterations in the hypothalamic–pituitary–adrenal (HPA) axis have also been reported in FM and other stress‐related disorders (Reyes del Paso et al. 2024), including associations with Monoamine Oxidase (*MAOA*) polymorphisms (Gürsoy et al. 2008) and FK506 Binding Protein 5 (*FKBP5*) rs1360780 variant (Hohne et al. 2015).

Neuroimmune dysregulation provides another relevant field. Polymorphisms such as Interleukin 6 (*IL6*) rs1800796 and Tumour Necrosis Factor (*TNF*) rs1800629 have been linked to elevated pro‐inflammatory activity, while Interleukin 10 (*IL10*) rs1800896 influences anti‐inflammatory responses (Fernández‐de‐las‐Peñas et al. 2022; Zlendić et al. 2024), although in FM it is unknown. Likewise, Interferon‐Induced Transmembrane Protein 3 (*IFITM3*) rs12252 has been studied in viral pathogenesis but not yet in FM (Fernández‐de‐las‐Peñas et al. 2024).

Despite increasing interest in the genetics of FM, most studies have focused on pain sensitivity, with limited attention to psychological dimensions. Considering the strong interaction between emotional distress and symptom severity, and the shared neurobiological substrates of pain and affective regulation, exploring genetic contributions to psychological traits is crucial.

This exploratory cross‐sectional study aims to examine associations between selected single nucleotide polymorphisms (SNPs) and psychological variables in women with FM. Identifying variants that contribute to maladaptive emotional responses may improve understanding of interindividual variability and support more personalised therapeutic strategies.

## 2 Methods

### 2.1 Study Design and Participants

A cross‐sectional observational study was conducted, in which standardised psychological and clinical assessments were collected from patients diagnosed with FM. The study adhered to the Strengthening the Reporting of Observational Studies in Epidemiology (STROBE) guidelines. The study was approved by the institutional ethics committee of the Universidad Rey Juan Carlos (with ID: 2605202012920), and all participants provided written informed consent prior to enrollment. The study adhered to the principles of the Declaration of Helsinki. Data were collected from December 2024 to May 2025.

The sample consisted of women diagnosed with FM according to the 2016 revision of the American College of Rheumatology (ACR) criteria residing in the Community of Madrid (CAM). Participants were recruited through the AFINSYFACRO patient association (Asociación de Fibromialgia y SFC, Functional Recovery Center for Patients with FM and CFS), as well as through announcements on bulletin boards and direct personal contact. All participants were interviewed and selected according to predefined inclusion and exclusion criteria. Inclusion criteria were: age between 18 and 65 years, a medical diagnosis of FM, pain duration longer than 12 weeks, and the ability to speak and understand Spanish fluently. The only exclusion criterion was the presence of cognitive impairment that prevented the participant from understanding or properly completing any of the outcome measures. Participants were selected via a non‐probabilistic convenience sampling method based on their availability and willingness to participate.

### 2.2 Outcome Measures

#### 2.2.1 Pain Intensity

Average Pain Intensity (API) at the time of assessment was recorded using a Numerical Rating Scale (NRS) ranging from 0 (no pain) to 10 (worst imaginable pain). This scale has demonstrated high validity and reliability in populations with chronic pain, including FM (Euasobhon et al. 2022).

#### 2.2.2 Fibromyalgia Impact

The Spanish version of the Fibromyalgia Impact Questionnaire (FIQ) was used to assess the impact of FM on physical function, pain, fatigue, sleep, and psychological well‐being. Scores range from 0 to 100, with higher scores indicating greater impact. It has been validated in Spanish populations with a sensitivity of 85.6% and a specificity of 73.2% (Rivera and González 2004).

#### 2.2.3 Psychological Variables

Anxiety was assessed using the State subscale of the State–Trait Anxiety Inventory (STAI‐S). This widely used questionnaire consists of 20 items rated on a 4‐point Likert scale, designed to evaluate transient emotional states of anxiety, which are influenced by situational and environmental factors. Higher scores indicate greater levels of state anxiety. The STAI‐S is one of the most frequently used tools in clinical and research settings to measure general anxiety levels (Julian 2011). The State subscale of the STAI was used in its validated Spanish version, which has demonstrated strong psychometric properties in both clinical and general populations (Buela‐Casal and Guillén‐Riquelme 2017).

Depressive symptoms were assessed using the Beck Depression Inventory (BDI). This instrument is widely used in both clinical and non‐clinical populations to evaluate the presence and severity of depressive symptoms. The Spanish version, used in this study and structurally equivalent to the original, consists of 21 items with 4 response options each. These items cover a broad range of cognitive, emotional, somatic, and behavioural symptoms associated with depression (Sanz 2005).

The Pain Catastrophising Scale (PCS) includes 13 items divided into three subdomains: rumination, magnification, and helplessness. Higher scores reflect greater catastrophising. The Spanish version has excellent internal consistency (ICC = 0.94) (García Campayo et al. 2008).

The Tampa Scale for Kinesiophobia (TSK) (11‐item version) measures fear of movement or re‐injury. Total scores range from 11 to 44, with higher values indicating greater kinesiophobia (Swinkels‐Meewisse et al. 2003). The TSK was applied in its validated Spanish version, which has shown good psychometric properties in populations with chronic pain (Gómez‐Pérez et al. 2011).

### 2.3 Selected Genetic Polymorphisms

This study employed a candidate‐gene approach to investigate the association between specific single nucleotide polymorphisms (SNPs) and psychological variables in fibromyalgia (FM). The selected SNPs were chosen based on their documented involvement in key biological pathways implicated in FM pathophysiology—including central pain modulation, stress response via the HPA axis, and immune‐inflammatory regulation—and their potential role in psychological vulnerability. These genes have been previously associated with pain sensitivity, emotional regulation, and neuroimmune interactions, which are central to the clinical expression of FM. Table 1 summarises the selected SNPs, their biological functions, and their relevance to FM and related pain conditions.

### 2.4 Genetic Analysis Procedure

Genetic material was obtained from saliva samples. Participants were instructed to refrain from eating, drinking or chewing gum for at least 1 h prior to sample collection. Saliva samples were collected, stored, and centrifuged at 3000 rpm for 15 min within 48 h of collection to isolate the cellular sediment, which was then stored at −20°C until analysis.

Genomic DNA was extracted from 500 μL of saliva using the MagMAX DNA Multi‐Sample Ultra 2.0 Kit (Thermo Fisher Scientific Inc., Hemel Hempstead, Hertfordshire, UK), following the manufacturer's protocol. DNA purification was performed automatically using the KingFisher Flex purification system (Thermo Fisher). DNA purity and concentration were assessed using the Quant‐iT PicoGreen dsDNA reagent (Thermo Fisher), and the resulting DNA was diluted to 5 ng/μL using 1× Tris‐EDTA (TE) buffer (Sigma‐Aldrich, Dorset, UK).

qPCR reaction mixtures of 10 μL contained 10 ng of genomic DNA as PCR template, 1× TaqMan Gene Expression PCR Master Mix, and 0.6× TaqMan SNP Genotyping Assay probe mix. The TaqMan SNP Genotyping Assays employed the 5′‐nuclease TaqMan chemistry to amplify and detect allele‐specific signals for each SNP in purified genomic DNA samples. The TaqMan Assay Predesigned SNP Genotyping Assay (Thermo Fisher Scientific Inc., Hemel Hempstead, Hertfordshire, UK) was also included. Each assay allows the genotyping of individuals for a single nucleotide polymorphism (SNP).

Real‐time PCR plates were processed at the university's technological support centers (CAT at URJC and/or Fundación Parque Científico de Madrid), under standard thermal cycling conditions (95°C for 10 min followed by 40 cycles of 95°C for 15 s and 60°C for 1 min). SNP variants were identified using specific fluorescent dyes.

### 2.5 Data Analysis

Data analysis was performed using the SPSS statistical software package (version 25.00). The Shapiro–Wilk or Kolmogorov–Smirnov tests were applied to assess the normality of variable distributions. Descriptive statistics were used to summarise the results: means, standard deviations, and ranges for parametric variables; and medians, modes, interquartile ranges, and ranges for non‐parametric variables. Correlational analyses between psychological and clinical variables were preceded by an assessment of each variable's distribution to determine the appropriate statistical test. Spearman's rho was used for variables that did not meet the assumptions of normality or had ordinal characteristics (pain intensity and fibromyalgia impact), while Pearson's correlation coefficient was applied to variables with approximately normal distributions and linear relationships (anxiety, depression, catastrophising and fear of movement).

For between‐group comparisons, one‐way analysis of variance (ANOVA) was applied for normally distributed variables, and the Kruskal–Wallis test was used for non‐parametric data. When significant differences were detected, post hoc analyses were conducted using Tukey's test for ANOVA and Mann–Whitney *U* tests for Kruskal–Wallis, as appropriate.

All single nucleotide polymorphisms (SNPs) were tested for Hardy–Weinberg equilibrium (HWE).

A 95% confidence interval was used, and *p*‐values less than 0.05 were considered statistically significant. To control for multiple comparisons, the False Discovery Rate (FDR) correction was applied using the Benjamini–Hochberg procedure, which offers a balance between Type I error control and statistical power, particularly suitable for exploratory studies involving multiple genetic variants. All *p*‐values presented in the tables are raw (uncorrected). However, only those associations that remained statistically significant after FDR correction were interpreted as meaningful in the results and discussion sections.

## 3 Results

### 3.1 Sociodemographic, Psychological and Genotypic Profile of the Sample

The sociodemographic characteristics of the sample (*n* = 67) were as follows:

The mean age was 57.50 ± 8.12 years. The mean weight was 74.48 ± 16.37 kg, and the mean height was 160.34 ± 21.03 cm. The calculated mean body mass index (BMI) was 28.97 ± 9.91 kg/m^2^. A summary of these data can be seen in Table 2.

**TABLE 2:** Sociodemographic and psychological characteristics of the sample. Values are expressed as mean ± standard deviation (SD).

Variable	Mean ± SD
Age (years)	57.50 ± 8.12
Weight (kg)	74.48 ± 16.37
Height (cm)	160.34 ± 21.03
BMI (kg/m^2^)	28.97 ± 9.91
API	7.02 ± 1.7
FIQ	71.26 ± 12.23
STAI‐S	39.57 ± 11.55
TSK	24.04 ± 6.06
PCS	25.19 ± 12.27
BDI	25.42 ± 10.98

Abbreviations: API = Average Pain Intensity; BDI = Beck Depression Inventory; BMI = Body Mass Index; FIQ = Fibromyalgia Impact Questionnaire; PCS = Pain Catastrophising Scale; STAI‐S = State subscale of the State–Trait Anxiety Inventory; TSK = Tampa Scale for Kinesiophobia.

Descriptive statistics for pain intensity, fibromyalgia impact, and psychological variables are likewise presented in Table 2. The API, measured using the NRS, was 7.02 ± 1.70, indicating substantial perceived pain. The FIQ showed a mean score of 71.26 ± 12.23, reflecting a significant functional impact on daily functioning. State anxiety (STAI‐S) averaged 39.57 ± 11.55, while fear of movement (TSK) was 24.07 ± 6.06. Pain catastrophising (PCS) had a mean of 25.04 ± 12.24, and depressive symptoms (BDI) averaged 25.42 ± 10.98, all indicating clinically relevant psychological distress in the sample.

### 3.2 Correlational Analysis Between Psychological Variables and Pain‐Related Self‐Report Measures in Fibromyalgia

The results revealed several significant associations between pain intensity, psychological distress, and functional impact. These relationships are summarised in Table 3, which presents the correlation coefficients and significance levels for all variables analysed.

**TABLE 3:** Correlation coefficients (*r*) and *p*‐values (*p*) for associations between pain intensity (API), fibromyalgia impact (FIQ), anxiety (STAI‐S), fear of movement (TSK), pain catastrophising (PCS) and depressive symptoms (BDI).

	API		FIQ		STAI		TSK		PCS		BDI	
	*r*	*p*	*r*	*p*	*r*	*p*	*r*	*p*	*r*	*p*	*r*	*p*
API	—	—	**0.493**	0.001*	**0.314**	0.009*	0.204	0.095	**0.414**	0.000*	**0.445**	0.000*
FIQ	**0.493**	0.001*	—	—	**0.360**	0.014*	0.027	0.861	0.198	0.188	0.276	0.069
STAI‐S	**0.314**	0.009*	**0.360**	0.014*	—	—	**0.329**	0.006*	**0.453**	0.000*	**0.416**	0.001*
TSK	0.204	0.095	0.027	0.861	**0.329**	0.006*	—	—	**0.445**	0.000*	**0.279**	0.023*
PCS	**0.414**	0.000*	0.198	0.188	**0.453**	0.000*	**0.445**	0.000*	—	—	**0.615**	0.000*
BDI	**0.476**	0.000*	0.276	0.069	**0.416**	0.001*	0.279	0.023*	**0.615**	0.000*	—	—

*Note:* Correlation tests were chosen according to variable distribution: Spearman's rho for non‐normal or ordinal measures (pain intensity, FM impact) and Pearson's coefficient for approximately normal variables (anxiety, depression, catastrophising, fear of movement). Coefficient > 0.60 indicated a strong correlation; coefficient between 0.30 and 0.60 indicated a moderate correlation; coefficient < 0.30 indicated a low correlation. Statistically significant correlations and *p* < 0.05 are marked (*). Raw *p*‐values are shown. FDR correction was applied to account for multiple testing. Only associations that remained significant after FDR correction were interpreted as statistically meaningful. Strong and moderate correlations are shown in bold.

Pain intensity (API) showed moderate positive correlations with fibromyalgia impact, catastrophising, and depressive symptoms. A weaker but significant correlation was observed between API and state anxiety with STAI‐S. FIQ was significantly correlated with STAI‐S but not with TSK, PCS, or BDI. Among psychological variables, PCS and BDI showed the strongest correlation, indicating a robust link between catastrophising and depressive symptoms. STAI–PCS and TSK–PCS also revealed moderate correlations, suggesting that anxiety and fear of movement are closely related to catastrophising tendencies. Additional significant associations were found between STAI–BDI and TSK–BDI, supporting the relationship between emotional distress and cognitive‐affective factors in FM.

### 3.3 Associations Between Pain Modulation Genes and Psychological Variables

The analysis of pain modulation genes revealed distinct patterns of association with psychological and clinical variables in patients with FM (Table 4).

**TABLE 4:** Mean scores (±SD) for psychological and pain related variables across genotypes of selected pain modulation genes. Genotype distributions are presented as absolute counts (*n*) and percentages (%).

*COMT* rs4680	Genotype	A/A	A/G	G/G	
	*n* (%)	13 (19.4%)	36 (53.7%)	18 (26.9%)	
	API	6.50 ± 2.10	7.33 ± 1.47	6.83 ± 1.86	*p* = 0.631
	FIQ	74.47 ± 10.84	69.61 ± 14.33	72.11 ± 7.90	*p* = 0.54
	STAI‐S	80.62 ± 27.77	79.09 ± 21.53	84.21 ± 20.10	*p* = 0.73
	TSK	22.38 ± 7.45	24.42 ± 5.68	23.67 ± 5.20	*p* = 0.592
	PCS	20.62 ± 12.94	25.31 ± 12.32	25.78 ± 10.77	*p* = 0.407
	BDI	26.50 ± 15.95	24.14 ± 10.37	25.29 ± 8.42	*p* = 0.86
*BDNF* rs6265	Genotype	C/C	C/T	T/T	
	*n* (%)	46 (68.7%)	18 (26.9%)	3 (4.5%)	
	API	6.97 ± 1.73	7.39 ± 1.75	6.00 ± 1.00	*p* = 0.283
	FIQ	72.89 ± 10.43	67.59 ± 15.64	74.67 ± 5.61	*p* = 0.604
	STAI‐S	78.33 ± 24.11	85.83 ± 16.86	94.50 ± 6.36	*p* = 0.33
	TSK	23.54 ± 6.09	24.22 ± 5.95	25.67 ± 2.08	*p* = 0.78
	PCS	22.43 ± 11.13	30.61 ± 12.95	20.00 ± 9.54	*p* = 0.044*
	BDI	24.45 ± 11.51	25.83 ± 9.53	25.33 ± 15.57	*p* = 0.94
*OPRM1* rs1799971	Genotype	A/A	A/G	G/G	
	*n* (%)	44 (65.7%)	18 (26.9%)	5 (7.5%)	
	API	7.00 ± 1.75	7.22 ± 1.62	7.40 ± 1.52	*p* = 0.86
	FIQ	70.59 ± 13.21	70.35 ± 10.35	75.55 ± 10.95	*p* = 0.16
	STAI‐S	75.67 ± 24.46	89.74 ± 14.04	94.50 ± 5.20	*p* = 0.029*
	TSK	23.64 ± 6.14	23.89 ± 5.92	24.00 ± 5.33	*p* = 0.875
	PCS	22.98 ± 11.53	27.78 ± 12.64	25.60 ± 11.66	*p* = 0.899
	BDI	24.24 ± 10.79	25.33 ± 11.12	26.60 ± 12.24	*p* = 0.936
*DRD3* rs6280	Genotype	C/C	C/T	T/T	
	*n* (%)	8 (11.9%)	27 (40.3%)	32 (47.8%)	
	API	7.50 ± 1.69	7.35 ± 1.60	6.66 ± 1.79	*p* = 0.16
	FIQ	69.10 ± 24.16	69.17 ± 11.38	73.92 ± 7.73	*p* = 0.34
	STAI‐S	79.75 ± 29.69	77.74 ± 24.06	83.70 ± 18.90	*p* = 0.60
	TSK	22.63 ± 8.38	24.11 ± 6.12	23.88 ± 5.12	*p* = 0.98
	PCS	23.25 ± 14.59	24.41 ± 12.89	24.94 ± 10.95	*p* = 0.90
	BDI	27.63 ± 14.98	25.50 ± 11.33	23.65 ± 9.75	*p* = 0.69

*Note:* Statistical comparisons were performed using one‐way ANOVA for variables with normal distribution and the Kruskal–Wallis test for non‐normally distributed variables. *p*‐values shown are raw. FDR correction was applied; significant results after correction are marked with *.

Abbreviations: API = Average Pain Intensity; BDI = Beck Depression Inventory; FIQ = Fibromyalgia Impact Questionnaire; PCS = Pain Catastrophising Scale; STAI‐S = State subscale of the State–Trait Anxiety Inventory; TSK = Tampa Scale for Kinesiophobia.

Significant associations were observed for two polymorphisms. The *BDNF* rs6265 polymorphism demonstrated a significant association with pain catastrophising (*p* < 0.05). Post hoc analysis using Tukey's test confirmed that individuals carrying the C/T genotype exhibited the highest mean PCS score, with the difference reaching statistical significance compared to both the C/C and T/T groups.

Similarity, the *OPRM1* rs1799971 gene showed a significant association with anxiety levels (*p* < 0.05), as measured by the STAI‐S. Participants with the A/G and G/G genotypes presented higher anxiety scores compared to those with the A/A genotype, suggesting a potential role of this polymorphism in modulating anxiety‐related traits in FM.

In contrast, the *COMT* rs4680 and *DRD3* rs6280 polymorphisms did not show statistically significant associations with any of the psychological or pain‐related measures assessed, including FIQ, STAI‐S, BDI, TSK and PCS.

### 3.4 Evaluation of Stress‐related Genetic Variants and Psychological Outcomes

The *MAOA* rs1137070 polymorphism showed a statistically significant association with depressive symptoms, as measured by the BDI (Table 5). Participants with the T/T genotype (*n* = 7, 10.4%) exhibited the highest mean BDI score (33.00 ± 15.73) with the difference reaching statistical significance (*p* = 0.02). Post hoc analysis confirmed that the T/T group differed significantly from both C/C and C/T genotypes, supporting the robustness of this association. No significant differences were observed across genotypes for other psychological or clinical variables, including API, FIQ, STAI‐S, TSK or PCS.

**TABLE 5:** Mean scores (±SD) for psychological and pain related variables across genotypes of selected stress‐mediated genes. Genotype distributions are presented as absolute counts (*n*) and percentages (%).

*MAOA* rs1137070	Genotype	C/C	C/T	T/T	
	*n* (%)	36 (53.7%)	24 (35.8%)	7 (10.4%)	
	API	6.92 ± 1.86	7.23 ± 1.63	7.00 ± 1.41	*p* = 0.67
	FIQ	69.38 ± 13.14	71.73 ± 11.42	79.18 ± 5.58	*p* = 0.76
	STAI‐S	41.28 ± 12.28	36.50 ± 11.31	38.71 ± 9.23	*p* = 0.29
	TSK	23.33 ± 6.50	24.88 ± 5.34	22.71 ± 4.39	*p* = 0.48
	PCS	23.53 ± 12.96	25.08 ± 11.41	27.71 ± 9.59	*p* = 0.40
	BDI	23.97 ± 9.80	23.78 ± 10.63	33.00 ± 15.73	*p* = 0.02*
*FKBP5* rs1360780	Genotype	C/C	C/T	T/T	
	*n* (%)	28 (41.8%)	30 (44.8%)	8 (11.9%)	
	API	6.66 ± 1.39	7.47 ± 1.81	7.38 ± 1.30	*p* = 0.08
	FIQ	70.40 ± 10.35	70.08 ± 15.53	79.04 ± 2.92	*p* = 0.135
	STAI‐S	40.57 ± 12.87	39.40 ± 10.32	37.25 ± 11.54	*p* = 0.89
	TSK	24.00 ± 5.19	23.87 ± 6.95	23.75 ± 4.33	*p* = 0.97
	PCS	24.39 ± 11.65	25.87 ± 12.85	22.25 ± 9.63	*p* = 0.83
	BDI	26.96 ± 12.81	25.30 ± 9.75	17.50 ± 6.33	*p* = 0.18

*Note:* Statistical comparisons were performed using one‐way ANOVA for variables with normal distribution and the Kruskal–Wallis test for non‐normally distributed variables. *p*‐values shown are raw. FDR correction was applied; significant results after correction are marked with *.

Abbreviations: API = Average Pain Intensity; BDI = Beck Depression Inventory; FIQ = Fibromyalgia Impact Questionnaire; PCS = Pain Catastrophising Scale; STAI‐S = State subscale of the State–Trait Anxiety Inventory; TSK = Tampa Scale for Kinesiophobia.

Regarding the *FKBP5* rs1360780 polymorphism, no statistically significant associations were found with any of the psychological or clinical variables assessed (Table 5). Although participants with the T/T genotype (*n* = 8, 11.9%) showed slightly lower BDI scores (17.50 ± 6.33) and higher FIQ scores (79.04 ± 2.92), these differences did not reach statistical significance.

### 3.5 Analysis of Immune and Inflammatory Gene Polymorphisms in Relation to Psychological Profiles

The analysis of immunomodulatory gene variants polymorphisms revealed limited evidence of statistically significant associations with psychological or clinical variables in FM patients (Table 6).

**TABLE 6:** Mean scores (±SD) for psychological and pain related variables across genotypes of selected immune modulatory genes. Genotype distributions are presented as absolute counts (*n*) and percentages (%).

*IL6* rs180079	Genotype	G/G	C/G	C/C	
	*n* (%)	51 (76.1%)	14 (20.9%)	2 (3.0%)	
	API	7.00 ± 1.41	7.00 ± 1.57	7.05 ± 1.80	*p* = 0.99
	FIQ	80.15 ± 13.36	72.10 ± 8.68	70.59 ± 13.21	*p* = 0.54
	STAI‐S	37.50 ± 2.12	43.29 ± 10.34	38.27 ± 12.16	*p* = 0.39
	TSK	23.00 ± 2.83	24.36 ± 4.24	23.71 ± 6.40	*p* = 0.91
	PCS	16.50 ± 10.61	26.57 ± 8.72	24.27 ± 12.84	*p* = 0.53
	BDI	18.00 ± 12.73	28.62 ± 10.28	24.18 ± 11.08	*p* = 0.31
*IL10* rs1800896	Genotype	C/C	C/T	T/T	
	*n* (%)	14 (20.9%)	28 (41.8%)	24 (35.8%)	
	API	6.82 ± 2.25	7.07 ± 1.56	7.13 ± 1.65	*p* = 0.99
	FIQ	71.16 ± 23.01	69.40 ± 9.43	74.48 ± 7.72	*p* = 0.53
	STAI‐S	40.93 ± 15.40	39.50 ± 12.14	38.79 ± 8.58	*p* = 0.35
	TSK	24.36 ± 4.57	25.04 ± 6.08	22.21 ± 6.28	*p* = 0.93
	PCS	27.29 ± 14.82	24.93 ± 9.37	22.88 ± 13.24	*p* = 0.53
	BDI	29.00 ± 14.12	25.75 ± 10.97	22.17 ± 8.91	*p* = 0.33
*IFITM3* rs12252	Genotype	A/A	A/G		
	*n* (%)	59 (88.1%)	8 (11.9%)		
	API	7.03 ± 1.79	7.13 ± 1.13		*p* = 0.84
	FIQ	69.98 ± 12.48	79.44 ± 5.12		*p* = 0.02*
	STAI‐S	38.29 ± 11.38	46.75 ± 12.25		*p* = 0.06
	TSK	23.69 ± 5.92	24.75 ± 6.02		*p* = 0.62
	PCS	24.34 ± 12.37	25.88 ± 9.69		*p* = 0.76
	BDI	24.41 ± 10.49	28.71 ± 15.07		*p* = 0.44
*TNF* rs1800629	Genotype	G/G	A/G		
	*n* (%)	57 (85.1%)	10 (14.9%)		
	API	7.06 ± 1.67	6.90 ± 2.08		*p* = 0.94
	FIQ	71.36 ± 12.87	71.57 ± 5.37		*p* = 0.732
	STAI‐S	40.21 ± 11.43	34.10 ± 12.64		*p* = 0.11
	TSK	24.05 ± 5.92	22.50 ± 5.89		*p* = 0.46
	PCS	25.60 ± 11.62	18.40 ± 13.09		*p* = 0.74
	BDI	25.51 ± 11.29	21.40 ± 8.96		*p* = 0.25

*Note:* Statistical comparisons were performed using one‐way ANOVA for variables with normal distribution and the Kruskal–Wallis test for non‐normally distributed variables. *p*‐values shown are raw. FDR correction was applied; significant results after correction are marked with *.

Abbreviations: API = Average Pain Intensity; BDI = Beck Depression Inventory; FIQ = Fibromyalgia Impact Questionnaire; PCS = Pain Catastrophising Scale; STAI‐S = State subscale of the State–Trait Anxiety Inventory; TSK = Tampa Scale for Kinesiophobia.

For *IL6* rs1800796, no significant differences were found across genotypes (G/G, C/G, C/C) in any of the variables assessed, including API, FIQ, STAI, TSK, PCS and BDI (all *p* > 0.05). Similarly, *IL10* rs1800896 genotypes (C/C, C/T, T/T) did not show significant associations with any of the psychological or clinical measures. In contrast, the *IFITM3* rs12252 polymorphism showed a statistically significant association with FIQ scores. Individuals with the A/G genotype had higher FIQ scores (79.44 ± 5.12) compared to the A/A group (69.98 ± 12.48), with the difference reaching statistical significance (*p* = 0.02). Finally, *TNF* rs1800629 did not show significant associations with any of the variables assessed (all *p* > 0.05).

## 4 Discussion

Despite the growing recognition of psychological distress in FM, research has historically prioritised clinical and somatic aspects—such as pain intensity and physical disability—over emotional and cognitive dimensions. The present study contributes to addressing this gap by examining the interrelationships among psychological variables and evaluating the role of genetic polymorphisms in these psychological traits.

In line with our correlational analysis, strong associations were found between pain catastrophising (PCS) and depressive symptoms (BDI), anxiety (STAI‐S), and kinesiophobia (TSK). Moreover, anxiety, depression, and fear of movement were also moderately interrelated, supporting the close interplay among cognitive and emotional factors in FM. These findings are consistent with previous research indicating that catastrophising acts as a central mediator between pain and emotional distress in FM (Ellingson et al. 2018; Lami et al. 2018; Riquelme‐Aguado et al. 2024). Moreover, the observed associations between anxiety, depression, and pain intensity reinforce the notion that psychological factors are not merely secondary consequences of chronic pain, but integral components of FM pathophysiology (Riquelme‐Aguado et al. 2024). The findings of Riquelme‐Aguado et al. (2024) suggest that emotional and cognitive symptoms may be involved in the perpetuation of neurogenic inflammation and central sensitisation, rather than being solely reactive to pain. These observations, together with the present results, underscore the relevance of considering cognitive‐affective processes in clinical assessment and in the design of comprehensive therapeutic strategies.

The present study also explored the genetic basis of these psychological traits, identifying a significant association between specific single nucleotide polymorphisms (SNPs) and psychological variables in FM patients. Although the differences in psychological scores between genotypes were modest, these findings offer preliminary evidence of a genetic contribution to psychological vulnerability in fibromyalgia, which may hold clinical relevance for future personalised approaches.

A significant association was observed between the *BDNF* rs6265 polymorphism and pain catastrophising. Specifically, participants with the C/T genotype showed higher levels of catastrophising compared to the C/C and T/T groups. This finding is consistent with previous studies (Xiao et al. 2012) suggesting that the presence of the Met allele (T) impairs *BDNF* secretion, alters synaptic plasticity, and contributes to increased emotional reactivity and vulnerability to stress. In FM, carriers of the T allele have been shown to display more pronounced symptomatology, especially in subgroups characterised by inflammatory and metabolic dysregulation (Nugraha et al. 2020). The present results suggest a possible involvement of BDNF not only in pain modulation but also in maladaptive cognitive responses such as catastrophising, which may play a role in the persistence of FM symptoms.

Although previous studies investigating the *BDNF* rs6265 polymorphism in FM have yielded mixed results regarding its diagnostic value (da Silveira Alves et al. 2020; Park et al. 2018; Polli et al. 2020; Xiao et al. 2012) the findings of this study suggest that BDNF may be related to psychological vulnerability in FM, rather than serving as a diagnostic marker. Although the association observed with pain PCS is noteworthy, no significant differences were found for other psychological domains, and the cross‐sectional design precludes establishing any modulatory role. The association between *BDNF* and catastrophising may reflect shared neurobiological mechanisms involving central sensitisation and emotional regulation, as BDNF is known to influence both nociceptive processing and affective reactivity (Polli et al. 2020).

The *OPRM1* gene encodes the μ‐opioid receptor, a key component in endogenous pain regulation and emotional processing (Tour et al. 2022). In the present study, findings revealed a significant association between the *OPRM1* rs1799971 and elevated anxiety levels, suggesting that this polymorphism may modulate affective responses in FM. Previous studies have shown that the G allele (A118G variant) may alter receptor binding affinity and reduce opioid efficacy, potentially impairing the natural inhibition of pain and stress responses (Mazzeo et al. 2023). Moreover, rs1799971 G/G genotype seems to confer higher susceptibility to FM (Estévez‐López et al. 2018), and its influence on anxiety‐related traits may reflect altered neurochemical signalling involved in emotional regulation.

The *MAOA* gene encodes monoamine oxidase A, an enzyme responsible for the degradation of neurotransmitters such as serotonin, dopamine, and norepinephrine (Shih et al. 1999). In our sample, the T/T genotype of *MAOA* rs1137070 was significantly associated with higher depressive symptoms, supporting its role in emotional dysregulation. This is consistent with prior evidence linking *MAOA* variants to increased risk of mood disorders and stress sensitivity in FM and other chronic pain conditions (Gürsoy et al. 2008). Given the central role of monoaminergic pathways in both pain and mood regulation (Bee and Dickenson 2009), *MAOA* polymorphisms may contribute to the affective burden of FM through impaired neurotransmitter metabolism and heightened stress reactivity.

Although *IFITM3* rs12252 did not show significant associations with psychological traits, it was linked to higher scores on the FIQ, suggesting a possible role in disease severity. *IFITM3* is involved in neuroimmune signalling and has been implicated in inflammatory responses and viral susceptibility (Clement et al. 2022; Gómez et al. 2021). Its relevance to FM may lie in its contribution to immune dysregulation and central sensitisation, which are increasingly recognised as key mechanisms in FM pathophysiology (García‐Domínguez 2025). While its direct impact on psychological symptoms remains unclear, the association with functional impairment warrants further investigation into its role in symptom amplification and systemic vulnerability.

Some of the analysed polymorphisms, including those in *COMT*, *FKBP5*, *DRD3*, *IL6*, *IL10* and *TNF*, did not show statistically significant associations with levels of anxiety, depression, pain catastrophising, or fear of movement. Although these genes are functionally relevant—given their roles in pain perception, stress regulation, and immune response—their influence on psychological dimensions of FM could not be confirmed in this sample (Braga et al. 2021; Martínez‐Jauand et al. 2013; Potvin et al. 2009; Wang et al. 2023).

This study supports a multifactorial model of FM in which genetic predispositions interact with psychological processes to shape symptom severity and disease impact. The influence of genes involved in pain modulation (*BDNF*, *OPRM1*), stress response (*MAOA*), and immune regulation (*IFITM3*) reflects the multifactorial nature of FM. These genetic pathways may be involved in the manifestation of psychological symptoms in patients with FM. While preliminary, these findings contribute to a better understanding of FM pathophysiology and suggest that integrating personalised approaches to psychological assessment and treatment could be valuable in future research and clinical practice.

## 5 Limitations

This study has several limitations. The exploratory nature of this study and the limited sample size restrict the generalisability of the findings. The sample size could be larger, as the current number of participants may have limited the statistical power to detect moderate or small genetic effects, especially in analyses involving less frequent genotypes. Additionally, no a priori sample size calculation was performed, since the number of participants was determined by the available funding for genetic analyses, which limited the sample size. Moreover, the cross‐sectional design restricts causal interpretations between genetic polymorphisms and psychological variables. Finally, this study employed a candidate‐gene approach focusing on a limited set of SNPs with documented roles in pain processing, stress regulation, and immune function. Although this targeted strategy is grounded in biological plausibility, it inherently limits the scope of genetic variability considered. Consequently, the design does not allow for genome‐wide exploration or the identification of novel loci, which may limit the comprehensiveness of the genetic associations observed. Overall, these findings provide preliminary evidence of associations between genetic variants and psychological factors in FM, but further longitudinal and mechanistic studies are required to elucidate the directionality and underlying pathways of these relationships.

## 6 Future Research Lines

Future research should aim to replicate these findings in larger and more diverse samples to increase statistical power and generalisability. Longitudinal designs would allow for a better understanding of how genetic variants influence the development and progression of psychological symptoms in FM over time. It would also be valuable to explore gene–environment interactions, particularly in relation to stress exposure, physical activity, and therapeutic interventions. Moreover, expanding the analysis to include other relevant genetic markers and combining genetic data with neuroimaging or neurophysiological measures could provide deeper insights into the biological mechanisms underlying cognitive‐affective dysregulation in FM. Integrating genetic profiles into personalised treatment approaches may ultimately help identify subgroups of patients who are more responsive to specific interventions.

## 7 Conclusions

This study highlights the relevance of psychological variables—particularly pain catastrophising, anxiety and depression—in the pathology of FM and explores their potential genetic underpinnings. Significant associations were found between the *BDNF rs6265* polymorphism and pain catastrophising, *OPRM1 rs1799971* and anxiety, and *MAOA rs1137070* and depressive symptoms. These findings provide preliminary evidence that specific genetic variants may be associated with emotional vulnerability and maladaptive cognitive responses in FM, highlighting the relevance of integrating psychological and genetic perspectives in future research and clinical approaches.

## Author Contributions

This study was designed by V.R.‐A. and G.D.‐G. The experiments were performed by V.R.‐A., M.E.G.‐A., S.D.‐B., L.R.‐L. and A.Z.C. M.E.G.‐A. and A.Z.C. were responsible for data curation. The data were analysed by A.G.‐C. and G.D.‐G., and the results were critically examined by all authors. V.R.‐A. and G.D.‐G. had a primary role in preparing the manuscript. F.G.‐E., M.E.G.‐A., S.D.‐B. and L.R.‐L. also contributed to the elaboration of the manuscript. All authors have approved the final version of the manuscript and agree to be accountable for all aspects of the work.

## Funding

This work was supported by Rey Juan Carlos University (Ayudas a Grupos de Investigación A639).

## Disclosure

The authors alone are responsible for the views expressed in this article. They do not necessarily represent the views, decisions, or policies of the institutions they are affiliated with.

## Conflicts of Interest

The authors declare no conflicts of interest.
